# Supplementary material for: Wnt5a Regulates Focal Adhesion Formation to Promote Migration in Ewing Sarcoma
Source: Cancers (Basel). 2025 Nov 20;17(22):3712. doi: 10.3390/cancers17223712 (PMC12650982; doi:10.3390/cancers17223712)
Supplement: Supplementary file 1 [file cancers-17-03712-s001.zip › cancers-3940342-supplementary.pdf]

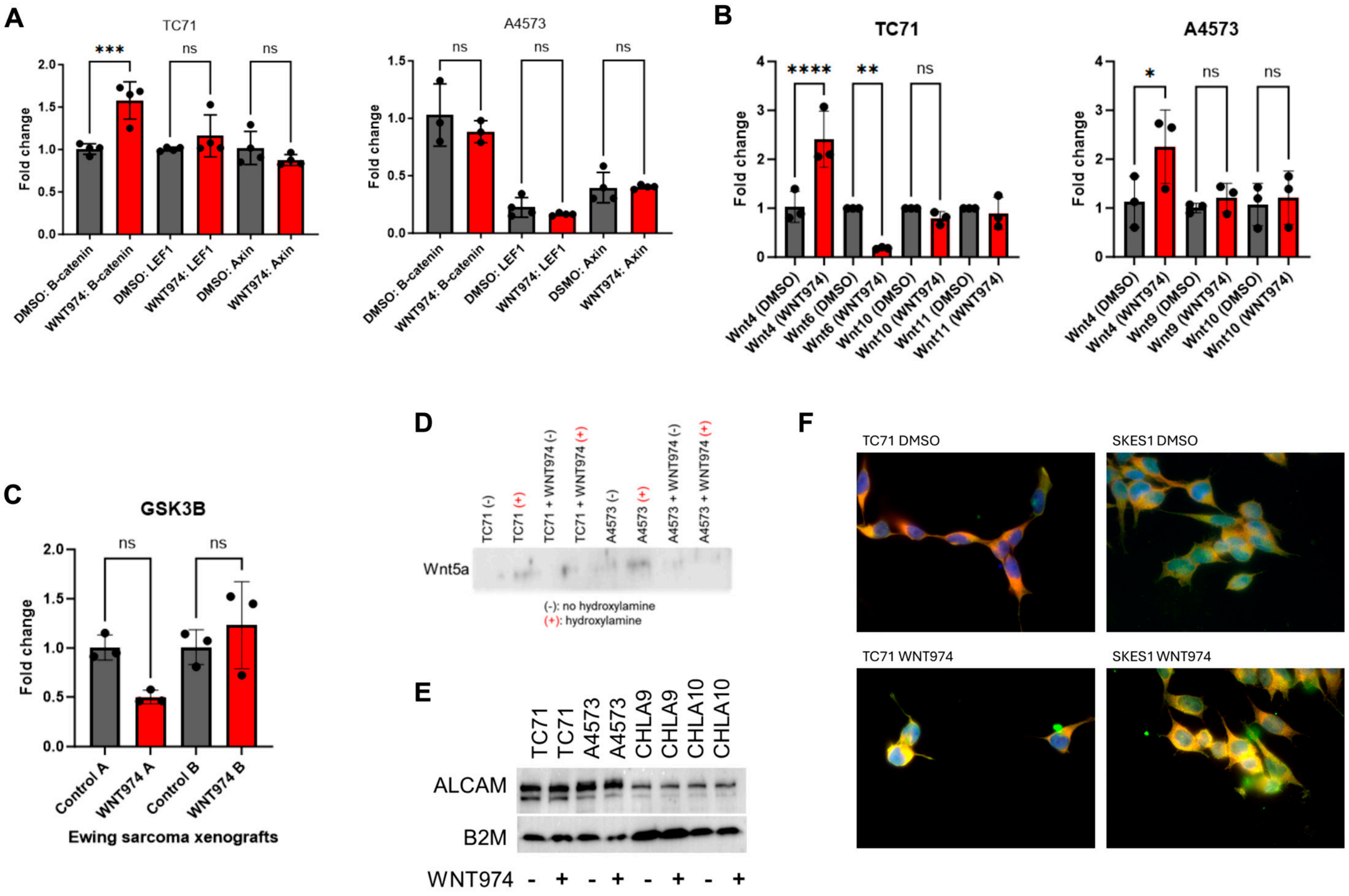
A
TC71
A4573
Fold change
***
ns
DMSO: B-catenin
WNT974: B-catenin
DMSO: LEF1
WNT974: LEF1
DMSO: Axin
WNT974: Axin
DSMO: Axin
B
TC71
A4573
****
**
*
Wnt4 (DMSO)
Wnt4 (WNT974)
Wnt6 (DMSO)
Wnt6 (WNT974)
Wnt10 (DMSO)
Wnt10 (WNT974)
Wnt11 (DMSO)
Wnt11 (WNT974)
Wnt9 (DMSO)
Wnt9 (WNT974)
C
GSK3B
Control A
WNT974 A
Control B
WNT974 B
Ewing sarcoma xenografts
D
TC71 (-)
TC71 (+)
TC71 + WNT974 (-)
TC71 + WNT974 (+)
A4573 (-)
A4573 (+)
A4573 + WNT974 (-)
A4573 + WNT974 (+)
Wnt5a
(-): no hydroxylamine
(+): hydroxylamine
E
TC71
A4573
CHLA9
CHLA10
ALCAM
B2M
WNT974
F
TC71 DMSO
SKES1 DMSO
TC71 WNT974
SKES1 WNT974

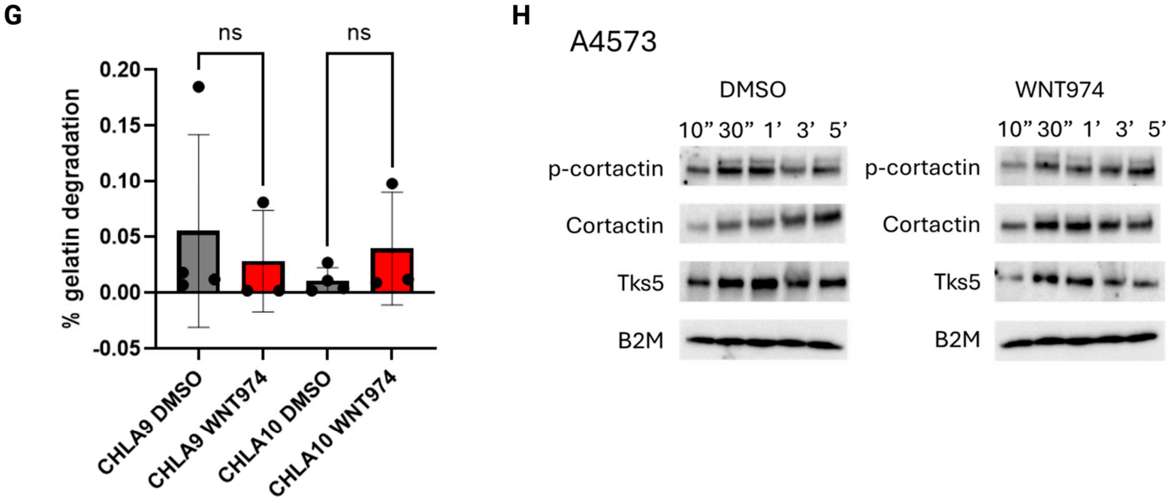
G
ns
ns
% gelatin degradation
0.20
0.15
0.10
0.05
0.00
-0.05
CHLA9 DMSO
CHLA9 WNT974
CHLA10 DMSO
CHLA10 WNT974
H
A4573
DMSO
WNT974
10” 30” 1’ 3’ 5’
10” 30” 1’ 3’ 5’
p-cortactin
Cortactin
Tks5
B2M
p-cortactin
Cortactin
Tks5
B2M

Figure S1

Supplementary Western Blot Figures

Figure 3A

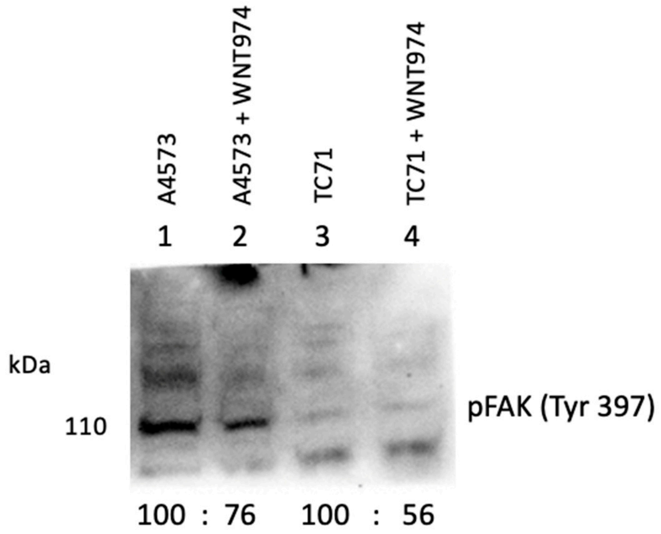

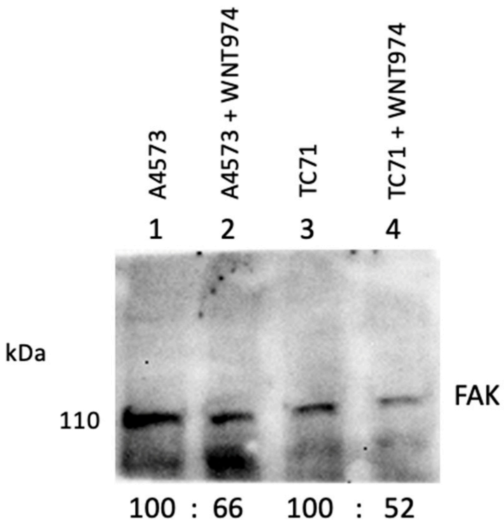

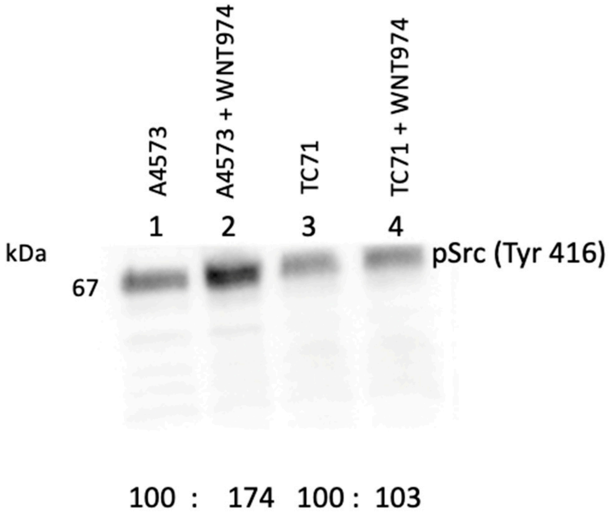

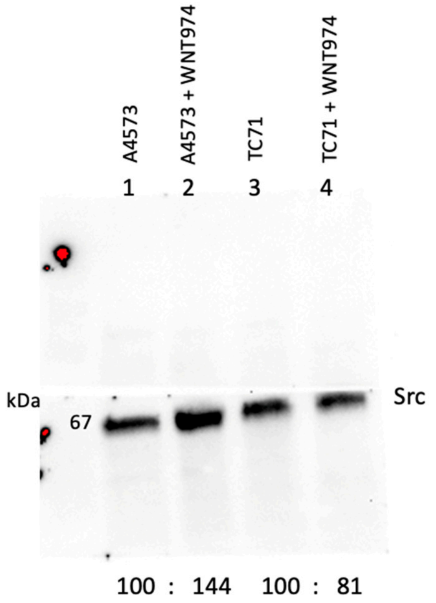

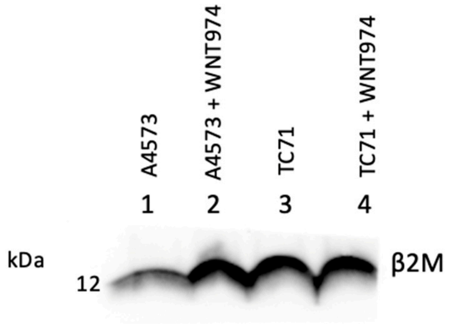

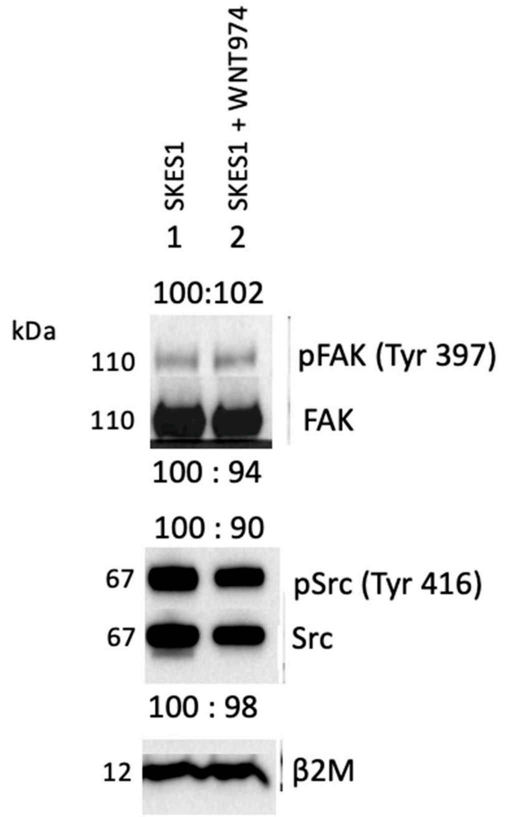
SKES1
SKES1 + WNT974
1
2
100:102
kDa
110
pFAK (Tyr 397)
110
FAK
100 : 94
100 : 90
67
pSrc (Tyr 416)
67
Src
100 : 98
12
β2M

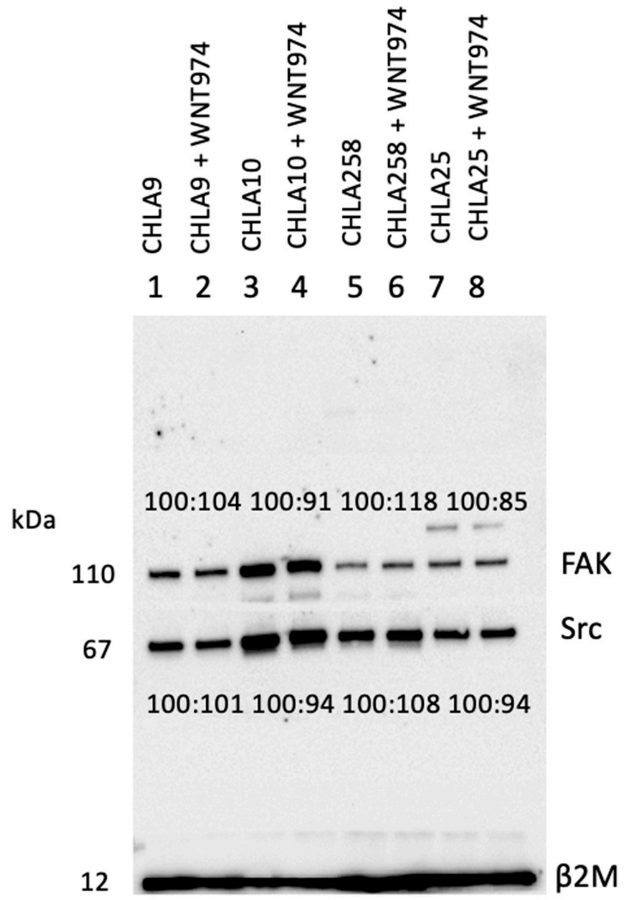
CHLA9
CHLA9 + WNT974
CHLA10
CHLA10 + WNT974
CHLA258
CHLA258 + WNT974
CHLA25
CHLA25 + WNT974
1 2 3 4 5 6 7 8
100:104 100:91 100:118 100:85
kDa
110
FAK
Src
67
100:101 100:94 100:108 100:94
12
β2M

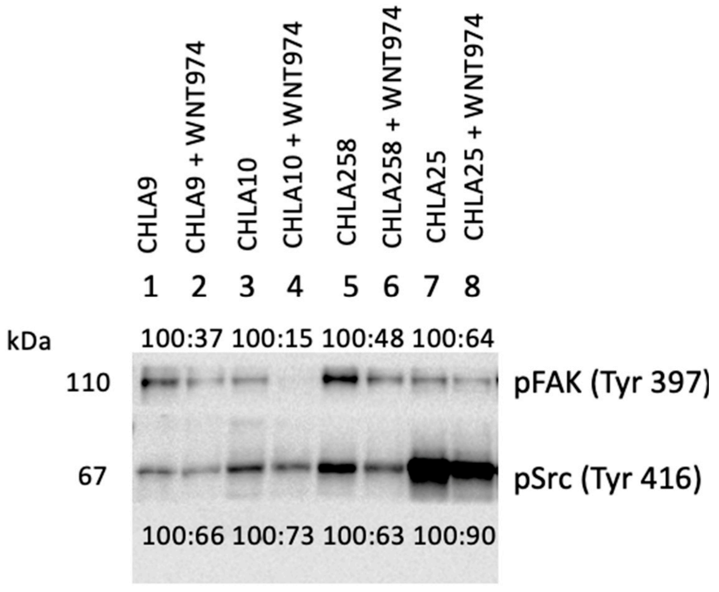
CHLA9
CHLA9 + WNT974
CHLA10
CHLA10 + WNT974
CHLA258
CHLA258 + WNT974
CHLA25
CHLA25 + WNT974
1 2 3 4 5 6 7 8
kDa
100:37 100:15 100:48 100:64
110
pFAK (Tyr 397)
67
pSrc (Tyr 416)
100:66 100:73 100:63 100:90

Figure 4C

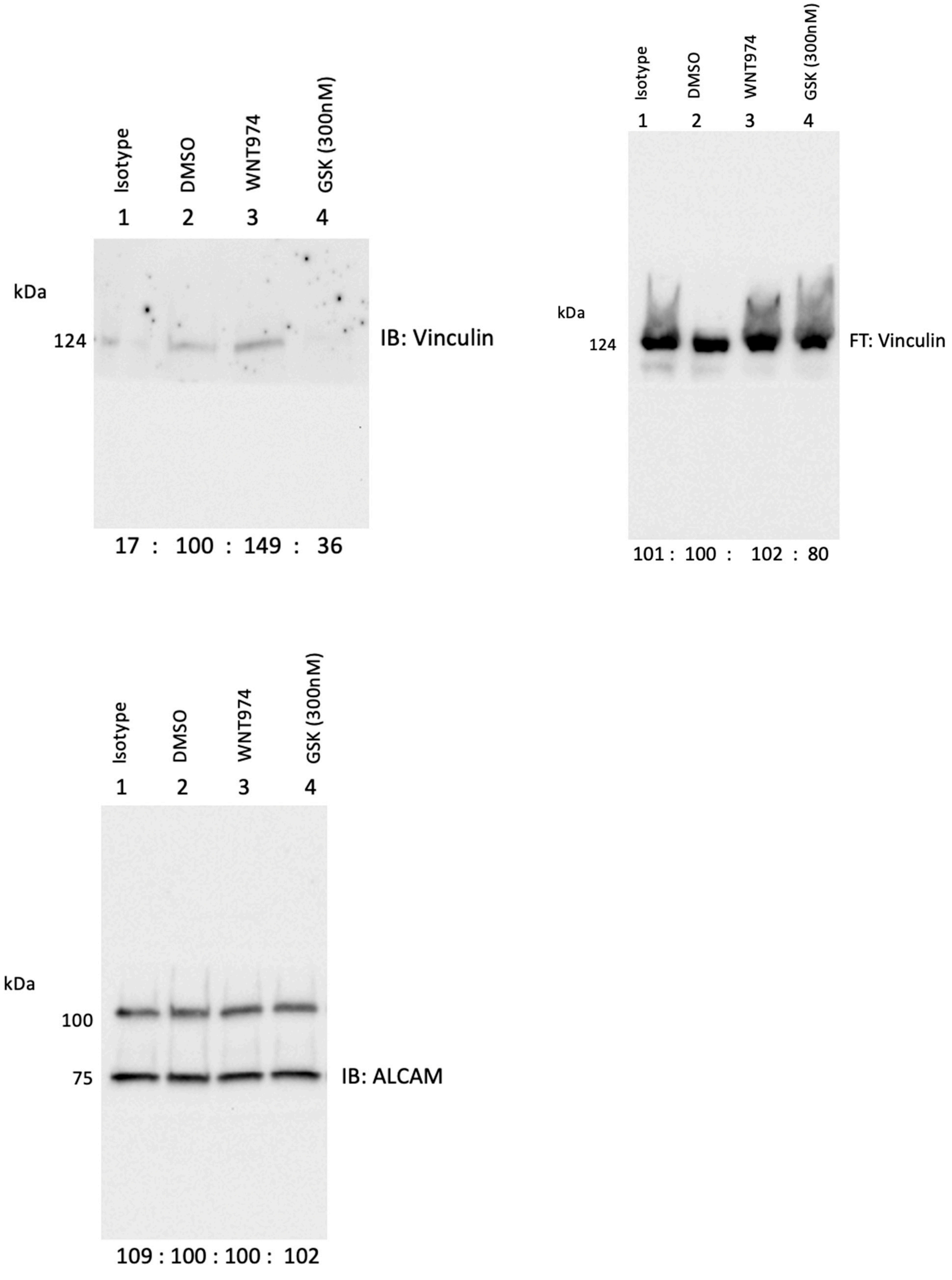

Isotype
DMSO
WNT974
GSK (300nM)
1
2
3
4
kDa
124
IB: Vinculin
17 : 100 : 149 : 36
Isotype
DMSO
WNT974
GSK (300nM)
1
2
3
4
kDa
124
FT: Vinculin
101 : 100 : 102 : 80
Isotype
DMSO
WNT974
GSK (300nM)
1
2
3
4
kDa
100
75
IB: ALCAM
109 : 100 : 100 : 102

Figure 5A

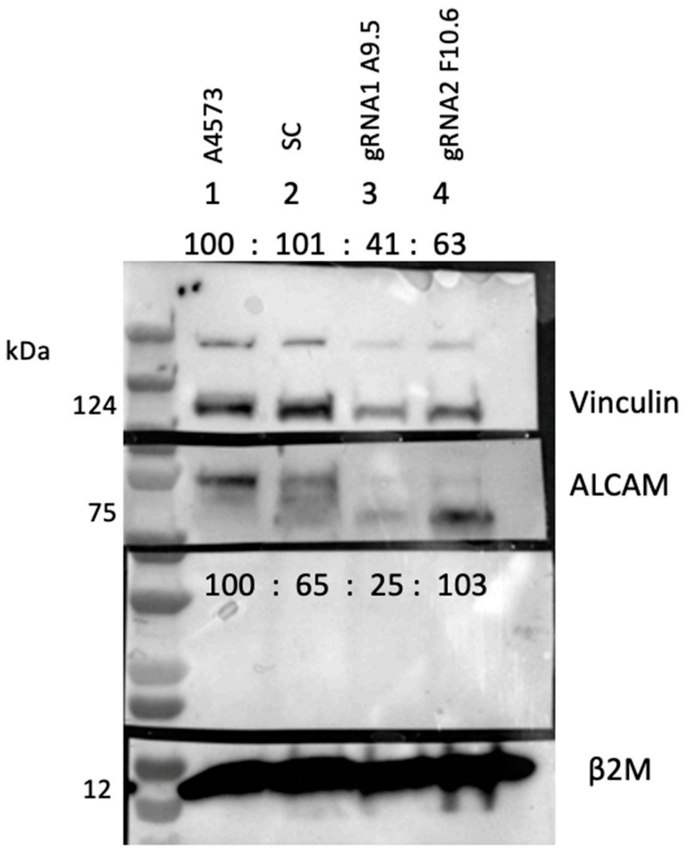
A4573
SC
gRNA1 A9.5
gRNA2 F10.6
1 2 3 4
100 : 101 : 41 : 63
kDa
124
75
12
Vinculin
ALCAM
100 : 65 : 25 : 103
β2M

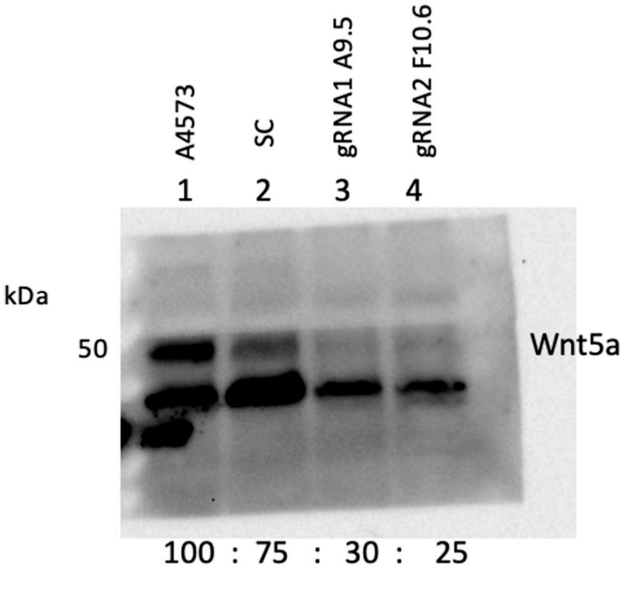
A4573
SC
gRNA1 A9.5
gRNA2 F10.6
1 2 3 4
kDa
50
Wnt5a
100 : 75 : 30 : 25

Figure 5F

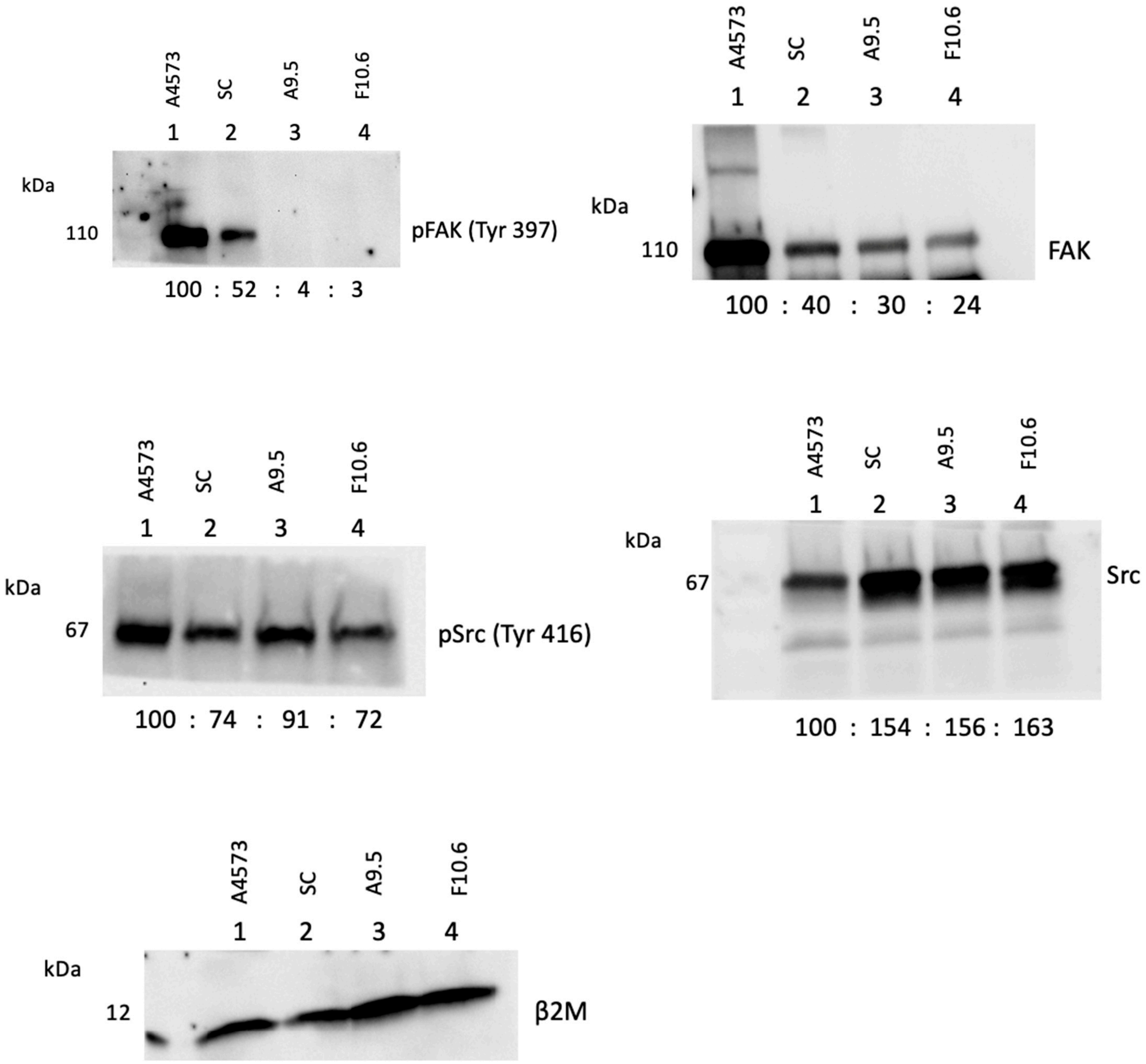
A4573
SC
A9.5
F10.6
1 2 3 4
kDa
110
pFAK (Tyr 397)
100 : 52 : 4 : 3
A4573
SC
A9.5
F10.6
1 2 3 4
kDa
110
FAK
100 : 40 : 30 : 24
A4573
SC
A9.5
F10.6
1 2 3 4
kDa
67
pSrc (Tyr 416)
100 : 74 : 91 : 72
A4573
SC
A9.5
F10.6
1 2 3 4
kDa
67
Src
100 : 154 : 156 : 163
A4573
SC
A9.5
F10.6
1 2 3 4
kDa
12
β2M

Supplementary Figure S1D

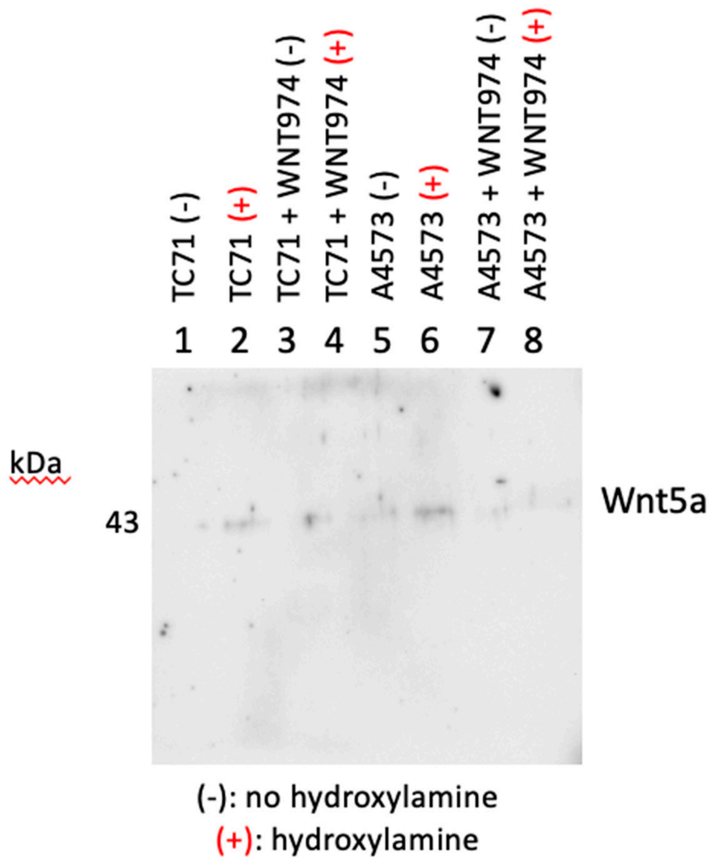

Supplementary Figure S1E

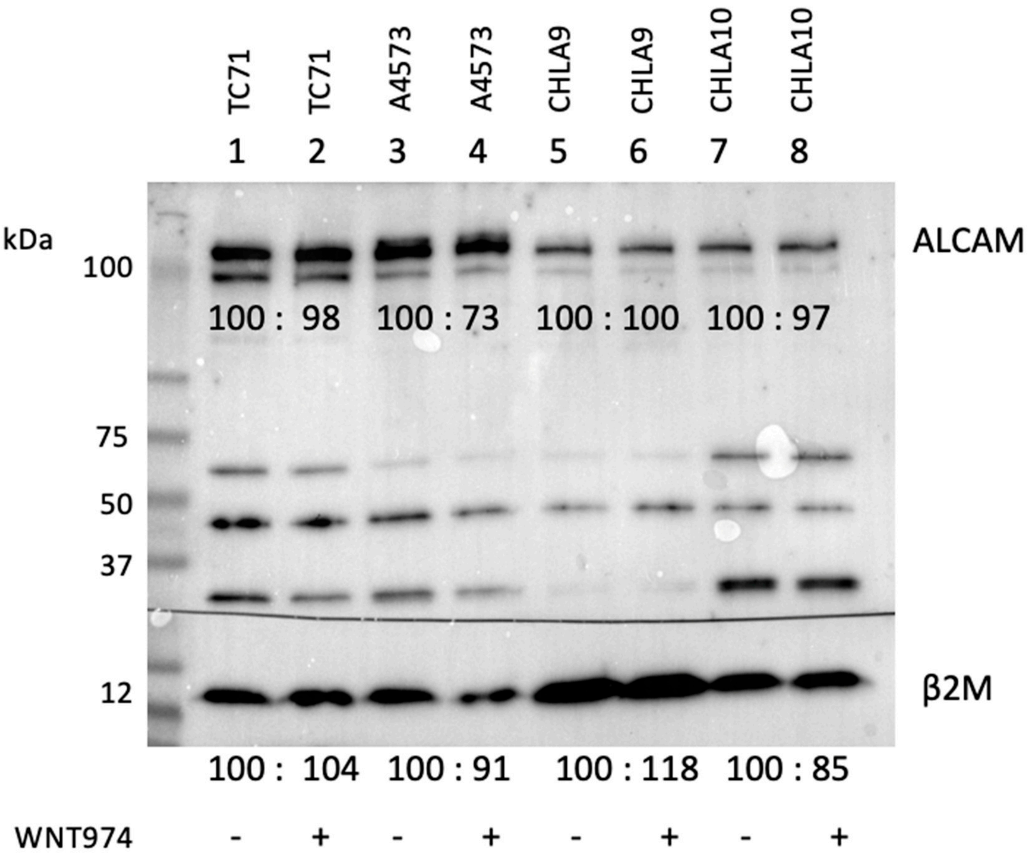

Supplementary Figure S1H

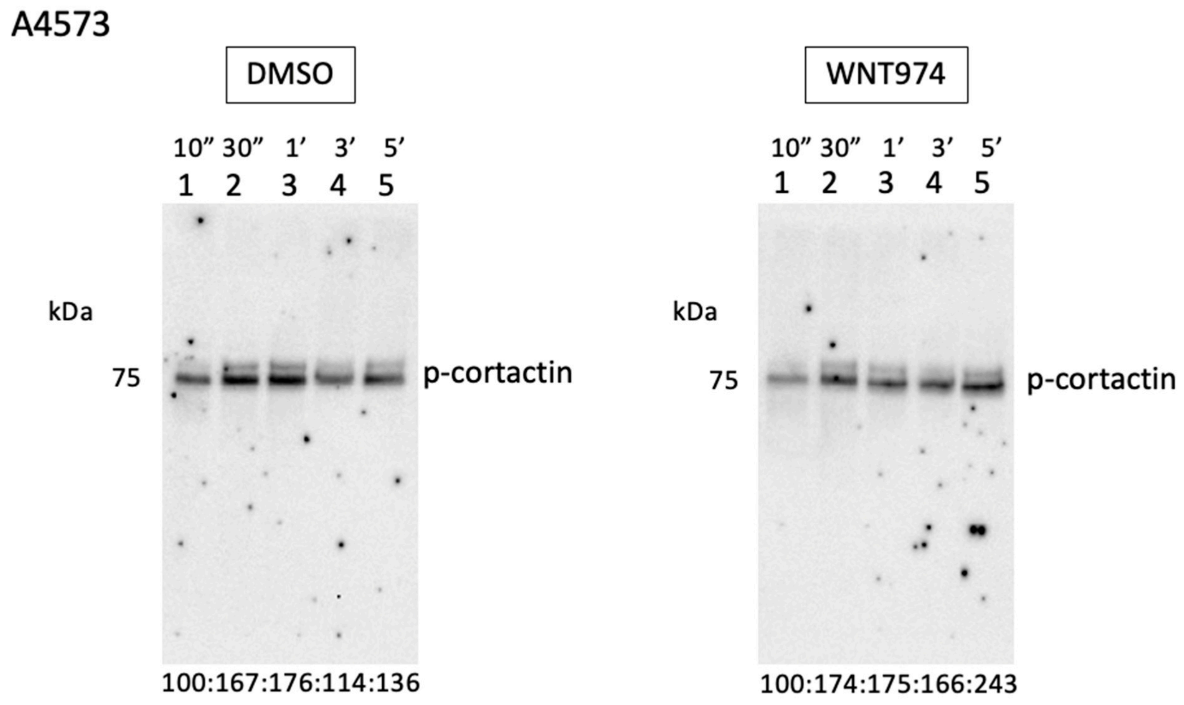

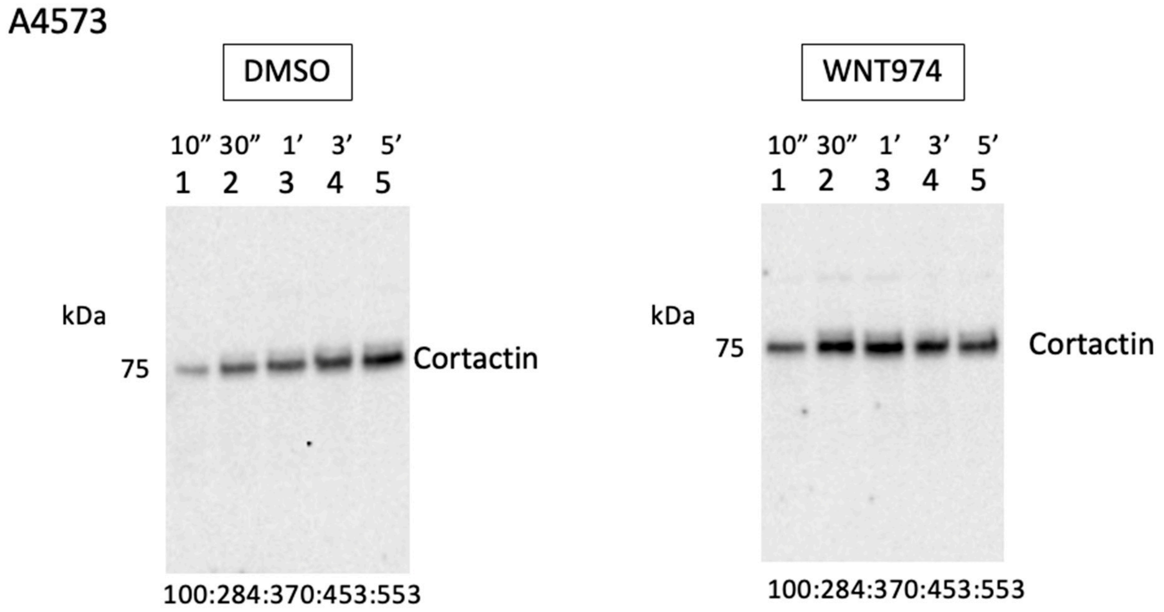

A4573

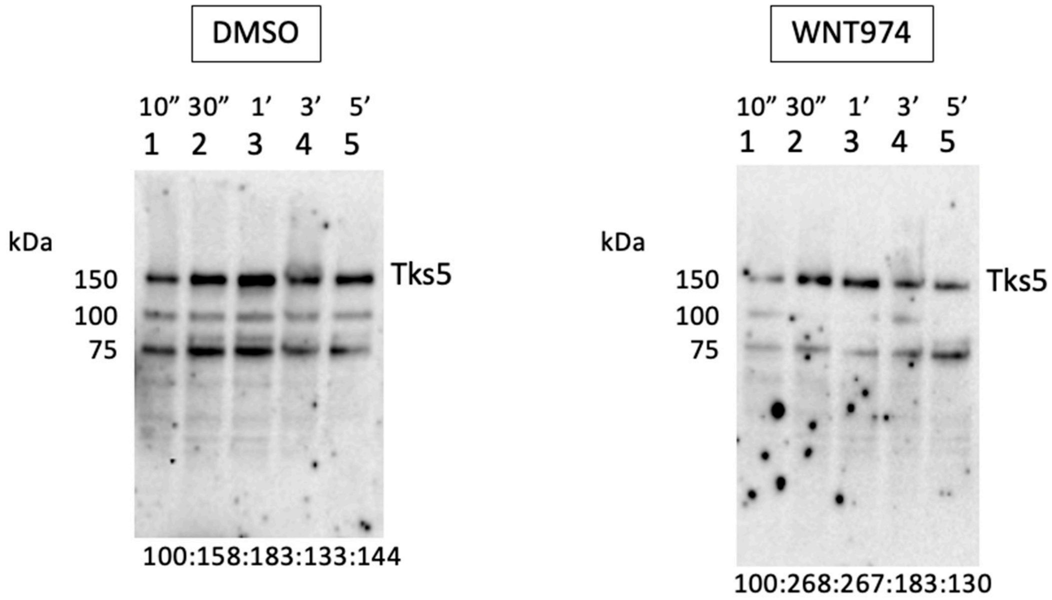
DMSO
10" 30" 1' 3' 5'
1 2 3 4 5
kDa
150
100
75
Tks5
100:158:183:133:144
WNT974
10" 30" 1' 3' 5'
1 2 3 4 5
kDa
150
100
75
Tks5
100:268:267:183:130

A4573

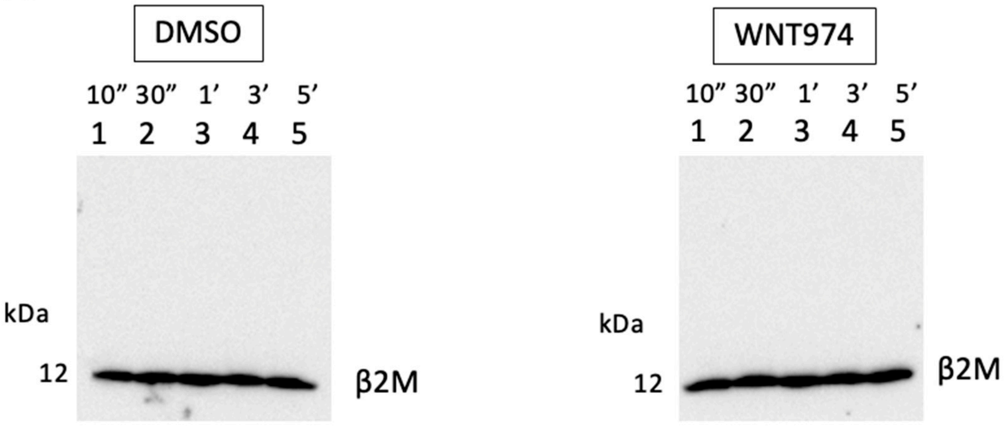
DMSO
10" 30" 1' 3' 5'
1 2 3 4 5
kDa
12
β2M
WNT974
10" 30" 1' 3' 5'
1 2 3 4 5
kDa
12
β2M
